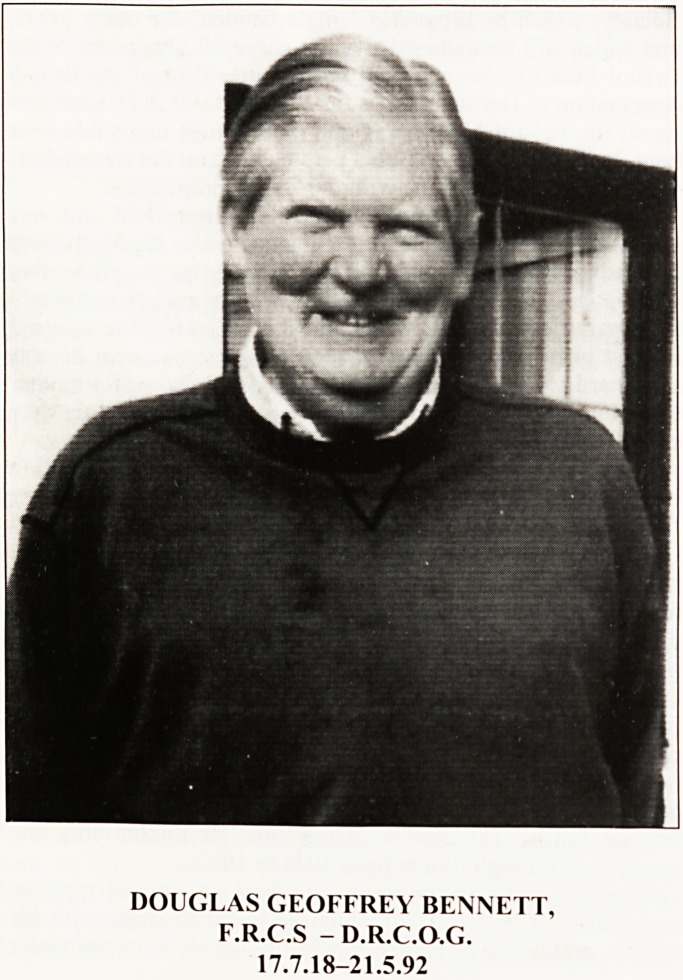# Dr D J Bennett

**Published:** 1992-12

**Authors:** 


### Obituary

**DOUGLAS GEOFFREY BENNETT,**
**F.R.C.S – D.R.C.O.G.**
**17.7.18–21.5.92**

Douglas Bennett, a general practitioner at Keynsham from 1935 to 1985, died in May 1992. He received his medical training at UCH and qualified MRCS, LRCP in 1942. The same year he entered the RAMC and served in S. Africa, India, Burma and Singapore where he was one of the first doctors to enter the notorious Japanese Prisoner of War "camp" at Changi Gaol after the liberation. On demobilisation he returned to UCH and then moved to Bristol as a surgical registrar. In 1952 he travelled the USA in company with his wife, visiting gynaecological and surgical centres. Having passed the FRCS in 1953 he decided to enter general practice and joined the practice in Keynsham where he continued for 35 years. With beds at Keynsham Hospital he was able to practice midwifery. He was also Clinical assistant to Mr. Bourns at the Bristol Royal Infirmary for many years and a member of a Boarding Panel for War Pensioners and Industrial Injuries.

His wife Marjorie was a consultant obstetrician and gynaecologist, they had a great love of Exmoor and for many years have had a thatched cottage at Porlock. In retirement they were able to enjoy to the full the country pursuits that Exmoor offers. Douglas followed the stag hounds and supported them as surgeon at point-to-points and local shows. He enjoyed rough shooting and particularly fishing at which he was especially skillful, making his own tackle and flies. He was an accomplished painter and made many pictures of the scenery he loved so well. He was a warm hearted man with a sense of fun and a great gift of friendship. He bore his last illness with his characteristic courage and cheerfulness. He is succeeded by his wife and daughter.

A.R.M.W., M.G.W.

## Figures and Tables

**Figure f1:**